# Sensors Based on the Carbon Nanotube Field-Effect Transistors for Chemical and Biological Analyses

**DOI:** 10.3390/bios12100776

**Published:** 2022-09-20

**Authors:** Yixi Deng, Lei Liu, Jingyan Li, Li Gao

**Affiliations:** 1 Department of Kidney Transplantation, The Second Xiangya Hospital of Central South University, Changsha 410011, China; 2 School of Life Sciences, Jiangsu University, Zhenjiang 212013, China

**Keywords:** carbon nanotubes, field-effect transistors, biochemical sensors, biomarkers

## Abstract

Nano biochemical sensors play an important role in detecting the biomarkers related to human diseases, and carbon nanotubes (CNTs) have become an important factor in promoting the vigorous development of this field due to their special structure and excellent electronic properties. This paper focuses on applying carbon nanotube field-effect transistor (CNT-FET) biochemical sensors to detect biomarkers. Firstly, the preparation method, physical and electronic properties and functional modification of CNTs are introduced. Then, the configuration and sensing mechanism of CNT-FETs are introduced. Finally, the latest progress in detecting nucleic acids, proteins, cells, gases and ions based on CNT-FET sensors is summarized.

## 1. Introduction

Effective diagnostic techniques are crucial for disease diagnosis and a timely follow-up treatment. However, most of the current disease diagnoses are based on the symptoms of the disease, but this is often not carried out in a timely manner or the disease is misdiagnosed because of the uncertainty of the symptoms [1]. Studies in genomics [2] and proteomics [3] illustrate that many new biomarkers, such as microions, viruses, DNA, proteins, peptides or cells, are closely linked to potential diseases. Therefore, a sensitive detection of these biomarkers can greatly improve the disease diagnosis and provide a more objective and quantifiable basis for clinical decision-making. However, most of the current detection methods, such as the enzyme-linked immunosorbent assay (ELISA) [4], the high-performance liquid chromatography (HPLC) [5] and the immunofluorescence assay (IFA) [6], have problems involving a lengthy consumption, complex steps, a low sensitivity, and radioactive pollution, which cannot be used to detect these trace substances. Moreover, some detection processes need secondary labeling, which has potential radiation hazards. This increases the cost and difficulty of detection and is not conducive to further improving the detection efficiency [7]. The advent of biochemical sensors provides a feasible solution for the rapid, sensitive and efficient detection and analysis of the disease-related microbiomarkers. Compared with the traditional detection methods, biochemical sensors are more and more widely used to detect various trace substances due to their advantages in offering a fast response, high sensitivity, strong specificity, a label-free detection and less sample demand [6,8,9,10].

**Figure 1:**
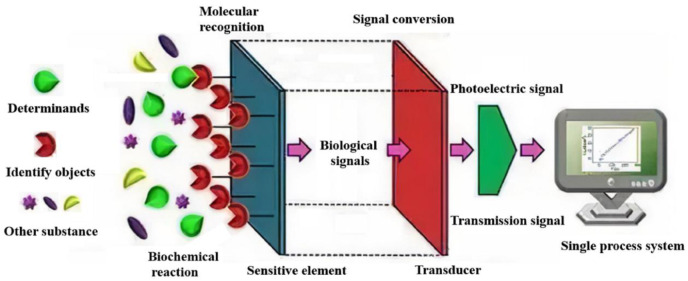
Schematic composition of biosensors.

A biochemical sensor is a device that combines the biological recognition elements with appropriate signal transduction elements for a reversible and selective detection of the concentration or activity of biochemical substances in various samples. The International Union of Theory and Applied Chemistry (IUPAC) defines it as an analytical device for converting biological reactions or biological components into useful signals, which is composed of a molecular recognition part (sensitive element) and a conversion part (transducer) [11,12], as shown in Figure 1. When the molecular recognition element is specifically combined with the detected substance, the biological signal is converted into an optical or electrical signal through the transducer (such as a point electrode, photosensitive tube, field-effect transistor, piezoelectric crystal, etc.). Finally, the target substance is detected and analyzed with the instrument. In addition, a biochemical sensor can be divided into biosensors and chemical sensors according to the detection substance. The biosensor is an instrument that is sensitive to biological substances and converts their concentration into electrical signals for detection. It is made of immobilized biological sensitive materials as recognition elements, including DNA, proteins, cells, etc., while chemical sensors are instruments that are sensitive to various chemicals and convert their concentrations into electrical signals for detection, such as gases, ions, etc.

Among the many types of biochemical sensors, field-effect transistor (FET) biochemical sensors have become the most widely used and with the fastest growing interest rate due to their real-time response and label-free detection [13,14]. When the transducer or recognition layer contains nanomaterials or nanostructures, the device is called a nanosensor. The development of new nanosensors provides a promising method for improving clinical diagnosis and treatment. People are increasingly interested in nanobiosensors [15,16,17,18].

The field-effect transistor (FET) biochemical sensor, based on nanomaterials, is a new type of biochemical sensor that was developed in recent years. On the one hand, this biochemical sensor has attracted the attention of many researchers due to its advantages of high miniaturization and high integration. On the other hand, nanomaterials have unique physical and chemical properties, such as a quantum effect, surface effect and micro-size effect [19]. Compared with the traditional detection technology, the sensor constructed by nanomaterials has the advantages of having a fast response, high sensitivity, good selectivity, lower reagent consumption, simple experimental operation and fast analysis speed, which are very suitable for the detection of biological ions and biological molecules [20,21,22,23]. Biochemical sensors, based on various nanomaterials, have shown a high sensitivity and selectivity to biomarkers, such as metal nanoparticles, quantum dots, nanowires, graphene, graphene quantum dots and carbon nanotubes [18,24,25,26,27], which can be specifically combined with the target analyte and detect its concentration and properties.

At present, the channel layer materials used to prepare field-effect transistor biosensors mainly include one-dimensional materials: carbon nanotubes (CNT) and silicon nanowires (SiNws), and two-dimensional materials such as graphene and molybdenum disulfide (MoS_2_).

A silicon nanowire is a typical one-dimensional nanomaterial. One of its advantages is that it has a large specific surface area and can produce a sharp response to the electric field. Silicon nanowires also have a good compatibility, can achieve a variety of biological receptors (such as peptide nucleic acid, aptamers, antibodies, etc.) and with direct surface modification, they have a strong potential for electronic applications. In addition, due to the small size characteristics of one-dimensional materials, the prepared biosensor can detect very low concentration markers in a small sample size. However, the fabrication process of nanowires is first to randomly grow silicon nanowires on the substrate material through a catalyst-assisted or self-assembly effects, and then assemble the silicon nanowires on the semiconductor silicon wafer [28]. The electrode fabrication and ohmic contact are completed by lithography, metal deposition and other processes. Therefore, the direction and size of the silicon nanowires prepared by this method on the substrate are difficult to accurately control, so the uniformity of SiNW is poor, resulting in an unsatisfactory stability and repeatability of the device. At the same time, the ‘bottom-up’ strategy is also limited by its complex preparation scheme, low success rate and lack of a reliable ohmic contact, which hinders any further application of the device [29].

Graphene is a two-dimensional semiconductor material with a zero band gap. The field-effect transistor prepared with graphene has attracted wide attention due to its high carrier mobility, low electrical noise, bipolar field-effect and good chemical and mechanical stabilities [30]. Piccinini et al. [31] prepared graphene using the drop coating method, and obtained a field-effect transistor biosensor with a cross-finger microchannel structure that could detect urea concentration, which has a higher sensitivity than the traditional electrochemical methods. Shin et al. [32] obtained a complete large area of graphene using chemical vapor deposition, then transferred it to a field-effect transistor and functionalized it to obtain a highly sensitive and selective glucose sensor. However, there is still a lack of simple methods of obtaining graphene without causing pollution and without the need for large areas. Furthermore, combined with the issue of a poor uniform performance, these are areas that require further research concerning the field of graphene field-effect transistor biosensors. These defects will seriously affect the preparation of the high sensitivity graphene field-effect transistor.

Molybdenum disulfide, based on a monolayer or multilayer two-dimensional hexagonal crystal structure, has excellent electrical properties, such as a high current on/off ratio, a large energy gap, a low threshold swing and a high mobility at room temperature, and it is a biocompatible material. It is widely used in the preparation of field-effect transistors and sensor devices [33]. Sarkar et al. [34] prepared a molybdenum disulfide layer by using the solution stripping method and transferred it to the channel position of the field-effect transistor. By silanizing the molybdenum disulfide layer, it is easier to modify the surface with biological probe molecules. The prepared field-effect transistor has an ultra-high sensitivity for pH detection and a wide pH operation range of 3~9. However, the large contact resistance between molybdenum disulfide and metal will seriously affect the performance of field-effect transistors, and it is difficult to accurately control the doping of two-dimensional materials through simple methods. Furthermore, the problem of molybdenum disulfide as a channel material still needs to be solved as it is difficult to grow high-quality ultra-thin metal oxides on the surface of the device as a gate dielectric layer.

In addition, the metal-organic frameworks (MOFs) with a porosity and conductivity are a new type of semiconductor materials with a high charge mobility and conductivity. The electronic band structure can be adjusted by ligand modification and metal selection, and the film can be prepared using a simple solution reaction, which can be used in the fields of electrocatalysis, fuel cells, supercapacitors and chemical sensing [35]. The MOF materials also have the advantages of having a high porosity, adjustable pore structure, diverse structure and surface modification, which are conducive to improving the sensitivity and response time of the sensor. In addition, the porosity and pore adjustability of MOFs can expand the application of the field-effect transistors in voltage-gated ion channels/microfluidic chips, and can greatly improve the sensitivity of gas/ion sensors based on field-effect transistors [36]. Therefore, the conductive MOFs can be used as an excellent choice for field-effect transistor active channel materials. However, most MOFs do not have any conductivity, which limits their application as electron transport materials in the electrical field [37].

Based on the limitations of the above materials as a special ballistic conductor and as an electron transfer medium, a MOF can speed up the transfer of electrons. It has a large specific surface area and a good biocompatibility. There are many active groups in the defect site, which can easily be functionally modified, so it can improve the fixation of chemical and biological molecules. Carbon nanotubes are used to modify the electrode with a high electrocatalytic activity, which can reduce the activation energy and the overpotential of the electrochemical reaction. It is suitable for improving the selectivity of a complex detection system by reducing the initial oxidation and detection peak potentials. These advantages make carbon nanotubes an ideal material for making field-effect tube biochemical sensors [38]. Therefore, this paper mainly introduces the properties and applications of field-effect tube biochemical sensors based on carbon nanotubes.

## 2. Biochemical Sensors Based on Carbon Nanotube Field-Effect Transistors

### 2.1. Carbon Nanotubes and Their Functional Modification

#### 2.1.1. Carbon Nanotubes

**Figure 2:**
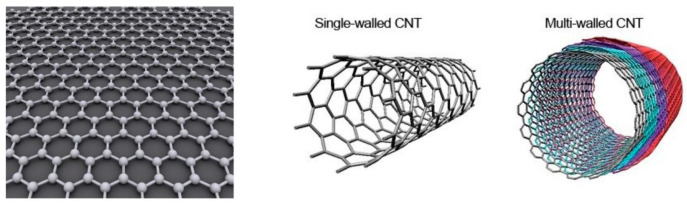
Basic structure of graphene (**left**), single-walled carbon nanotube (**middle**) and multi-walled carbon nanotube (**right**, the layers between the MWNTS are distinguished by different colors). Reproduced from Gookbin et al. [44] by permission of Molecular Diversity Preservation International.

Carbon nanotubes (CNT), also known as the Pakistani tube, are nano-materials with a hexagonal honeycomb lattice composed of sp^2^ carbon units [25], which were discovered by the Japanese scientist, Professor Iijima in 1991 [39]. The CNT can be regarded as the curling composition of graphene sheets with an axial symmetry, which can be divided into single-walled carbon nanotubes (SWCNTs) and multi-walled carbon nanotubes (MWCNTs) according to the layers of the graphene sheets [40], as shown in Figure 2. Due to their unique structural characteristics, such as a large specific surface area, a good biocompatibility, a high conductivity, an excellent electrocatalytic activity and a high chemical stability, carbon nanotubes can promote the transfer of electrons, making them an excellent electrode modification material with good metal or semiconductor properties. They have been widely used in supercapacitors, catalyst carriers, catalysts, drug delivery, sensors and other fields [41]. In terms of energy conversion, carbon nanotubes can be used as a carrier of DNA molecular probes to promote the electron transfer between electroactive species and electrodes. Therefore, carbon nanotubes are widely used in electrode modification to construct electrochemical sensing platforms [42]. Due to their atomic thickness, excellent electrical properties, good biocompatibility and dimensional compatibility, they are an ideal channel material for constructing ultra-sensitive FET biochemical sensors [43].

The diameter of a SWNT is usually between 0.5 nm and 2 nm, while its length can vary between several orders of magnitude (from 10 nm to 1 cm). Its length is important for some applications, but the electronic properties of SWNTs depend on the direction in which the graphene is rolled up to form nanotubes. This direction is called the chiral vector. Each carbon atom in graphene can be identified by a pair of integers (n, m) and a pair of lattice vectors (a_1_, a_2_), thus obtaining the definition of the chiral vector Ch = na_1_ + ma_2_. The size of the chiral vector determines the diameter, d = C_h_/π. At room temperature, about 67% of the single-walled carbon nanotubes are semiconducting and 33% are metallic. For semiconductor single-walled carbon nanotubes, the band gap is inversely proportional to its diameter. Moreover, the optical transitions of metal and semiconductor single-walled carbon nanotubes mainly depend on the variations of their diameters and chiral vectors. Therefore, in order to obtain uniform electrical and optical properties, the monodisperse nanotubes need to be monodispersed in their diameter and electronic type, because nanotubes of almost the same diameter may have different chiral vectors and thus have different electronic properties. In addition, single-walled carbon nanotubes with the same chiral support can have a different chiral orientation, which will affect the interaction between single-walled carbon nanotubes and circularly polarized light [45].

Usually, synthesized single-walled carbon nanotubes are a mixture of metal tubes and semiconductor tubes, and for many applications, the presence of unwanted types of single-walled carbon nanotubes affects the optimal performance [46]. For example, in the field-effect transistor, the metal single-walled carbon nanotubes will hinder the effective electronic switch. The separated semiconductor single-walled carbon nanotubes (S-SWCNT) improve the on/off switching ratio of the field-effect transistor [47] and the separated metal single-walled carbon nanotubes (M-SWCNT) perform best in transparent conductive films [48,49]. In these cases, the selective removal of unwanted SWNT types is sufficient. Therefore, the separation of these different substances is particularly important for improving the performance of SWCNT-based electronic devices. Many methods have been reported to separate S- and M-SWCNTs from synthetic bulk materials [46]. For example, metal carbon nanotubes can be converted into semiconductor carbon nanotubes if treated with diazonium salts or carbodichloride. This is attributed to the fact that the diazo covalent functionalization significantly disturbs the electronic and optical properties of metal single-walled carbon nanotubes, it affects the availability of electrons near the Fermi level in metal single-walled carbon nanotubes, and it stabilizes the charge transfer state before bonding. Following the covalent functionalization with p-hydroxybenzene diazonium salt, a negative charge is induced on the metal single carbon nanotube by deprotonation in an alkaline solution, allowing the subsequent electron type separation using free solution electrophoresis [50]. Similarly, dichlorocarbobenzene opens an energy gap at the Fermi level, converting the metal single-walled carbon nanotubes into semiconductors [51]. However, the separated SWCNTs are rarely used for biochemical and chemical sensors, possibly due to the high material cost and the difficulty in removing the dispersant used in the purification process [52]. For example, SWCNTs can be easily separated on a large scale by coating the SWCNTs with π-conjugated polymers, but this method also removes the polymers from SWCNTs except for a few examples using supramolecular polymers [53].

#### 2.1.2. Preparation Methods of the Carbon Nanotubes

The reported preparation methods of carbon nanotubes mainly include the graphite arc method, the flame method, the chemical vapor deposition method, the laser evaporation method, the template method, the in-situ synthesis method and the pyrolysis polymer method [54,55]. The most commonly used are the arc discharge and the chemical vapor deposition.

##### Laser Evaporation

Laser deposition is a vacuum physical deposition process. Firstly, the catalyst is mixed with graphite to prepare the graphite target. Then, the graphite target is put into the high-temperature resistant quartz tube, and the inert gas is heated to 1200 °C. The high temperature and high-pressure gaseous carbon are produced by laser burning. These gaseous carbon and catalyst particles are oriented to the local expansion emission, and the CNTs are deposited on the substrate under the action of the catalyst. However, this method is difficult to further apply due to its relatively high cost [56].

##### Arc-Discharge 

The arc discharge method is to apply a potential difference of about 20 V between two graphite electrodes 1–3 mm apart in an inert atmosphere (usually argon). The generated arc discharge makes the graphite evaporate from the anode and it condenses at the cathode to form carbon nanotubes. When using a pure graphite electrode, the multi-walled carbon nanotubes will be produced. Adding metal catalysts (such as iron, nickel or cobalt) to the anode can lead to the SWCNT formation. However, the purity of carbon nanotubes cannot be controlled by the arc discharge, and further operation is needed to obtain pure carbon nanotubes [57].

##### Chemical Vapor Deposition

Chemical vapor deposition (CVD) is currently the most viable method for the industrialized high-quality and large-area graphene preparation. Graphene is prepared using the CVD method at 800~1200 °C. The gas is introduced into a reaction chamber with catalyst particles, and the gas is decomposed and deposited on the template to generate carbon nanotubes. The advantage of the CVD method is that the reactant is gas and can leave the reaction system after the reaction. Therefore, the purity of carbon nanotubes is high, but the diameter of the prepared carbon nanotubes is not uniform, and the shape is irregular [58,59].

#### 2.1.3. Functional Modification of Carbon Nanotubes

Covalent, non-covalent and binding modifications of metal nanoparticles are all types of functionalization for carbon nanotubes.

**Figure 3:**
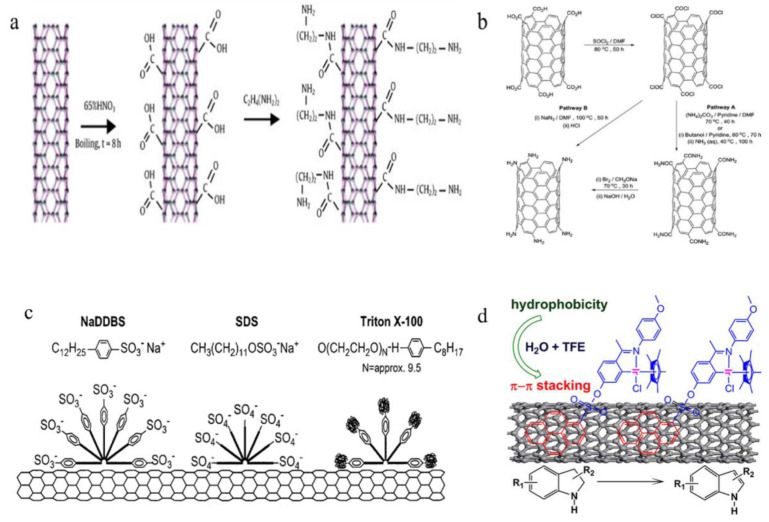
Covalent (**a**,**b**) and the non-covalent modifications (**c**,**d**) of carbon nanotubes. (**a**) Carboxyl functionalized carbon nanotubes. Reproduced from Phuong et al. [63] by permission of Elsevier Science Ltd. (**b**) Amino functionalized CNT. Reproduced from Campidelli et al. [64] by permission of Bentham Science Publishers.; (**c**) Iridium metal complex catalyst was coated on the surface of the carbon nanotubes through a non-covalent bond accumulation. Reproduced from Islam et al. [67] by permission of the American Chemical Society; (**d**) Diagram of the surfactant adsorption on the CNT surface. Reproduced from Cui et al. [68] by permission of Tech Science Press.

The covalent modification mainly involves the chemical disruption of C-C bonds at the CNT’s ports or side walls, resulting in the generation of polar carboxyl or hydroxyl groups on their surfaces. Subsequently, various target products containing specific functional groups, such as chemical groups, fluorescent labeled molecules, DNA, anticancer drugs and other substances, are attached to CNTs through derivative reactions [60,61,62]. Figure 3a illustrates how Tam et al. produced amine groups in ethylenediamine after introducing -COOH-derived groups on the surface of CNTs using an oxidation reaction [63]. Nitric acid, mixed acid (concentrated sulfuric acid/nitric acid), neutral hydrogen peroxide and sodium hydroxide are frequently employed oxidants for the covalent modification. However, a covalent modification may potentially compromise the structural integrity of CNTs and affect their mechanical and electrical characteristics. As shown in Figure 3b, Campidelli et al. prepared an amino-functionalized SWCNT using the Hofmann rearrangement of carboxylate amides (pathway A) and using the Curtius reaction of carboxylate chloride with sodium azide (pathway B) [64].

The non-covalent modification refers to the modification of carbon nanotubes by non-covalent bonding, such as the physical adsorption and surface coating, without introducing covalent chemical bonds. The non-covalent modification mainly occurs through the hybridization of carbon atoms sp^2^ in side-wall graphene structures to form highly delocalized electrons and generate non-covalent bonds with electrons of other compounds [65,66]. A non-covalent interaction mainly includes the dispersion force, the hydrogen bond, the dipole-dipole force, the π-π stacking effect and the hydrophobic effect. All of the carbon atoms in the carbon nanotubes are sp^2^ hybrids. These atoms are prone to forming highly delocalized electrons and can be combined with other π-electron-rich compounds by π-π stacking. In addition, compared with the covalent modification, the non-covalent modification of carbon nanotubes has a complete structure and can maintain its original performance, as shown in Figure 3c. Mohammad et al. coated carbon nanotubes with sodium benzoate and sodium dodecyl benzene sulfonate to increase their water solubility [67]. The non-covalent modification substances mainly include surfactants, molecules, polymers containing aromatic groups, etc. As shown in Figure 3d, Wang et al. coated the surface of carbon nanotubes with the metal iridium complex catalyst through a non-covalent bond accumulation, with a coating efficiency of over 94%. The metal iridium complex catalyst was coated on carbon nanotubes, and the indole dehydrogenation reaction was catalyzed by methanol, ethanol, tetrahydrofuran, trifluoroethanol and other organic compounds. Then, the charge repulsion caused them to disperse, when attached to their surface by the carbon nanotubes. Under thermodynamics, water-soluble polymers such as sodium polystyrene sulfonate tangle carbon nanotubes, act as surfactants and make them amphiphilic substances [68]. For biochemical sensors, the non-covalent modification of carbon nanotubes can not only improve their water solubility in biological systems, but also avoids the non-specific adsorption of biomolecules. The non-covalent functionalization of carbon nanotubes has been considered the most suitable for fabricating photosystem-based biochemical sensors and has been widely used in DNA sensors [69,70,71,72].

The binding modification of metal nanoparticles refers to the combination of carbon nanotubes with some metal nanoparticles, so as to produce synergistic effects in electrical connections, which can further improve sensitivity [73,74,75]. Dilonardo et al. used Au NPs with a controllable size and deposition to modify the CNT surface, which enhanced the interaction between the gaseous analyte and the sensing layer, thus improving the sensitivity of NO_2_ gas [76].

### 2.2. Biochemical Sensors Based on the Carbon Nanotube Field-Effect Transistors 

The concept of a field-effect transistor (FET) was first mentioned in Lilienfeld’s patent, in 1928. In 1947, Bell et al. prepared the first point contact field-effect transistor made from a germanium semiconductor [77]. The field-effect transistor is a kind of triode semiconductor device which can achieve the control of the current by changing the internal electric field of the semiconductor. Its basic structure includes the source, drain and gate triode structure and the semiconductor channel layer between the source and the drain (the effect of this layer is that the dielectric layer can control the current in the channel layer when the gate voltage is applied).

A field-effect transistor sensor is based on the interaction between the tested compounds and the FET conductive channel, which induces the change of channel surface charge transfer, doping and scattering effect, and the change of the interface capacitance and the Schottky barrier, resulting in the change of channel conductance and then successfully completing the detection of target molecules. It has the advantages of having a high sensitivity, an easy miniaturization and a real-time detection [78,79]. At the same time, the field-effect transistor sensor has a signal amplification effect. When a gate voltage V is applied between the source and the drain electrodes, the electrical signal of the sensing channel will increase or decrease the geometric multiple. Therefore, the sensor’s sensitivity can be improved by applying the gate voltage. The field-effect transistor sensor can amplify the electrical signal and change the direction of the current. Therefore, the typical field-effect tube sensor has the Dirac point, namely the inflexion point of the current direction change. The Dirac point changes after the interaction between the conductive sensitive material and the target. Therefore, the corresponding relationship between the concentration of the detection substance and the Dirac point can also be used to achieve the quantitative detection of the target [80].

Field-effect transistor sensors can be divided into back gate field-effect tube sensors and solution gate field-effect tube sensors, according to the position of the grid voltage. The back gate field-effect transistor sensor means that the gate, the source and the drain are not on the same side, and it does not need to grow a gate dielectric layer. The preparation process is relatively simple, which can reduce the damage to the channel layer material caused by multiple processing. The solution gate field-effect tube sensor applies the gate voltage Vg to the solution above the sensing channel, but not in contact with the sensing substrate and electrode materials. The gate voltage applied by the back gate field-effect transistor sensor is much larger than that of the solution gate field-effect transistor.

Field-effect transistor biochemical sensor, also known as the semiconductor biochemical sensor, is based on the structure and working principle of a metal-oxide-semiconductor-field-effect transistor (MOS-FET). The device replaces the metal gate in the MOS-FET structure with the ion-sensitive membrane or the ion-sensitive membrane and electrolyte solution or the reference electrode modified by biological elements (antibodies or antigens, etc.) [81]. In other words, the biomolecule recognition receptors, such as the monoclonal antibodies or single-stranded DNA (ssDNA) probes, are modified on the surface of the FET, which can selectively bind to the biomolecules in the solution environment. When the charged biological molecules recognize and form complexes on the ion-sensing membrane, or the biological molecules undergo biochemical reactions on the ion-sensing membrane to form ionic products, the charge density on the surface of the ion-sensing membrane changes, thereby changing the potential of the ion-sensing membrane, which is equivalent to adjusting the gate voltage through the external power supply to control the channel current between the source electrode and the drain electrode [77]. Moreover, a linear correlation exists between the biomolecule adsorption amount on the sensitive gate membrane and the drain output current within a certain range. Therefore, the biological reactions on the gate ion-sensitive membrane can be quantitatively analyzed by measuring the leakage current, including the nucleic acid hybridization, the protein interaction, the antibody-antigen binding, and the enzyme-substrate reaction [82,83].

Carbon nanotubes have the characteristics of a nanometer size, a huge specific surface area and a surface effect. When a specific molecule is adsorbed on the surface of the carbon nanotubes, it will bend the energy band of the carbon nanotubes and affect their electronic structure, thus leading to changes in the transport properties of carbon nanotubes. This change provides the possibility for carbon nanotubes as sensitive materials. Single-walled carbon nanotubes (SWCNTs) can be divided into metal-type and semiconductor-type. Based on the resistance response characteristics of semiconductor carbon nanotubes to adsorbed chemicals, chemical or biological sensors based on field-effect transistors are prepared [84]. When chemical molecules or biological materials are adsorbed on the surface of semiconductor carbon nanotubes, it will lead to an electron transfer and change its conductivity, which makes carbon nanotubes an ideal sensor material. Multi-walled carbon nanotubes (MWCNTs) have a multi-layer tubular structure compared with single-walled carbon nanotubes, and the former have a more complex chemical adsorption mechanism. The conductivity of multi-walled carbon nanotubes is less sensitive than that of single-walled carbon nanotubes because of the lack of a carbon band gap or narrow band gap. Because multi-walled carbon nanotubes are usually metal types, the influence of the chemical adsorption on the conductivity is not obvious. However, related studies have shown that multi-walled carbon nanotubes have excellent sensing properties for water vapor [85,86] NH_3_ [87,88], NO_2_ [89,90] and O_2_ [91,92].

Although different carbon nanotube field-effect transistors consist of different structures, they all contain similar structures: conductive channels, source, drain and gate electrodes at the top or bottom of the channel, as well as a dielectric layer that separates the gate and carbon nanotube between the channel and the gate [93]. The working principle is similar: the gate electrode uses a vertical electric field to control the charge in the channel. The horizontal electric field between the source electrode and the drain electrode provides the driving force, so the current flows from one electrode to another through the CNT [94]. 

**Figure 4:**
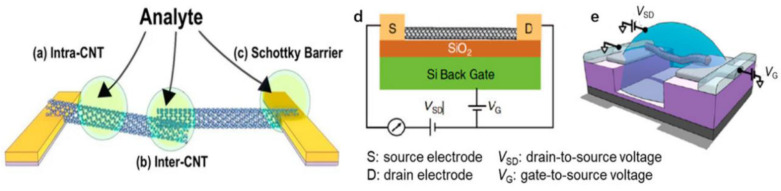
A schematic diagram of the sensitive sites that may affect conductivity (**a**–**c**), a schematic diagram of carbon nanotube field-effect transistors for sensing: (**a**) at the side wall or along the length of the CNT itself, (**b**) the interface between the CNT and CNT (between the CNT) and (**c**) the interface metal electrode and the CNT (Schottky barrier) between the CNT. Reproduced from Moghaddam et al. [96] by permission of the American Chemical Society; (**d**) General CNT—FET. Reproduced from Sharf et al. [97] by permission of Elsevier Science Ltd.; (**e**) Electrolyte-gated CNT—FET. Reproduced from Sharf et al. [98] by permission of the American Chemical Society.

In the nano biochemical sensor based on the CNT-FET, single-walled carbon nanotubes show great advantages. Paul et al. [95] reported the application of a single-walled carbon nanotube field-effect transistor in gas sensors, pointing out that the sensing mechanism of a single CNT-FET or a thin-film CNT-FET is mainly derived from the gas adsorption on the carbon nanotubes and the metal modulation of the Schottky barrier at electrodes. Simply put, the response to the analyte is attributable to changes in one or all of the conduction characteristics of the following three components of the device [96]. As shown in Figure 4a–c, the length of the transmission along the tube shows that the contact point between the tubes is a tunnel junction, and the contact point between the tube and the metal electrode is a Schottky barrier.

The modulation of the Schottky barrier is caused by the change of the work function of the electrode metal or CNTs in the presence of the target analyte. The modulation between the CNTs corresponds to the change of the transmission coefficient of the tunnel junction between the tubes, which can be attributed to the change of distance between the tubes or the change of work function of the tubes. The conduction modulation in the CNT is caused by the change of charge density along the side wall (leading to a doping effect) or the carrier scattering characteristics along the side wall (affecting mobility). Each of these three modulations may affect the global device response, depending on the type of steering and the device configuration [99]. 

Figure 4d shows the typical configuration of a CNT-FET for sensing purposes. Generally, the carrier transport in a CNT-FET can be attributed to four states, independent of the device structure, mainly depending on the length of the CNTs and the average free path length of the CNTs and the contact type between CNTs and the source and drain electrodes [96]. For example, the ohmic contact ballistic CNT-FET refers to the carrier injected into the carbon nanotubes from the source and drained through the ohmic contact. The carrier transport process in the carbon nanotubes is not subjected to any scattering.

In contrast, the Schottky diffusion-type CNT-FET means that the carrier injection is affected by the Schottky barrier from the electrode and the CNT’s heterojunction. The carrier continuously scatters during the transmission in the conductive channel. The carriers can be divided into two types: holes and electrons. If the carrier type is mainly electrons, then the field-effect transistor is an n-type transistor, and if the carrier is mainly holes, then the FET is a *p*-type transistor. Theoretically, the metal-carbon nanotube contact depends on the work function difference between the metal electrode and the carbon nanotube. Still, the *p*-type CNT-FET is more common due to the influence of the physical and chemical properties of the electrode.

In addition, the electrolyte-gated FET biochemical sensors have attracted extensive attention due to the advantages of easy processing, low cost, good sensitivity, biocompatibility and low working voltage [100,101,102,103]. The typical configuration of electrolyte-gated CNT-FETs is shown in Figure 4e. The electrolyte is used to replace the dielectric material to contact the gate electrode and channel directly [98]. The biggest difference between the working principle of the electrolyte gate-controlled field-effect transistor and that of the traditional field-effect transistor is that the gate electrode adjusts the channel current through the electrolyte solution. The biggest advantage is that the electrolyte’s giant double electron layer effect can make the sensor obtain the same current with a smaller gate voltage, and it can usually work at a very low voltage (1 V) [104,105,106]. Some extreme electrochemical reactions can be avoided, such as the decomposition of water and the destruction of biological activities [107]. Therefore, it is commonly used to detect biological samples in a solution environment.

#### 2.2.1. Biosensors Based on the CNT-FET for DNA Detection

Using DNA information to diagnose and treat diseases at the molecular level is extremely important for precision medicine. Through the comprehensive analysis of DNA gene sequences, valuable medical information can be obtained, conducive with the early prevention of diseases. A DNA biosensor based on the SWNT-FET has great potential in large-scale gene detection, clinical diagnosis and rapid detection of environmental monitoring. DNA can be accumulated on the surface of nanotubes through the nucleic acid base π-π [108,109], and a single SWNT is wrapped by the aromatic interaction between the nucleic acid-base and the side wall of the SWNT, thus forming a stable hybrid with a single SWNT [110].

The nucleic acid analyte (DNA) was effectively combined with a CNT-FET biosensor. The DNA detection by sequence-specific hybridization is a common detection strategy to ensure the specificity of biosensors. The FET biosensor can combine the complementary DNA, RNA or peptide nucleic acid (PNA) chains with the sensor’s surface and then make it specifically bind to the chain to be tested to ensure its specificity and generate detectable electrical responses. The recent research on DNA biosensors based on a carbon nanotube field-effect transistor is summarized in Table 1. 

**Figure 5:**
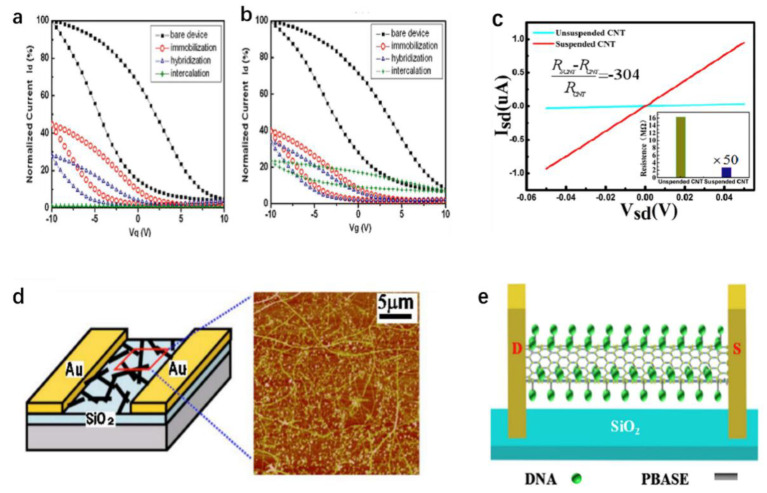
(**a**) Hybridized with a complementary target analyte; (**b**) Typical gate voltage dependence of a normalized drain current DS hybridized with a single-base mismatch target analyte; (**c**) Comparison of the electrical conductivity between the suspended CNT and the unsuspended CNT. In the illustration, ×50 represents the 50-fold amplification of the resistance of the suspended CNT. Reproduced from sun et al. [115] by permission of Elsevier Science Ltd.; (**d**) Device schematics and atomic force microscope images. Reproduced from Gui et al. [124] by permission of the American Institute of Physics; (**e**) Schemata of a SCNT-FET.

Dekker et al. [123] covalently linked the carboxylated SWNT tip with a charge-free DNA analogue-coupled peptide nucleic acid (PNA) and then detected the hybridization of PNA-DNA by atomic force microscopy (AFM). This work laid the foundation for the subsequent development of the SWNT-based biosensors. Star et al. [111] used a carbon nanotube field-effect transistor (CNT-FET) as a selective detector for the DNA immobilization and hybridization, confirming that the immobilized CNT-FET attached to oligonucleotides can specifically recognize target DNA sequences, such as SNPs responsible for hereditary hemochromatosis. Gus et al. [124], using the terminally modified NH_2_ oligomers (NH_2_-DNA) immobilized on SWNT-FET, could stably detect and distinguish complementary and single-base mismatch DNA chains with oligomers and improved their sensitivity using thread embedding agents. Figure 5 shows the schematic diagram of the carbon nanotube device and typical atomic force microscope images showing single-chain carbon nanotubes and catalyst particles on the device. Figure 5 shows that the transition curves between NH_2_-DNA modified SWNT-FETs and bare devices are compared. Compared with the single-base mismatch hybridization, the current (Ids) detected by the SWNT-FET with the target DNA complementary hybridization decreases more obviously. This phenomenon shows that the double-stranded DNA formed by complementary hybridization may increase the scattering center on the semiconductor channel or the work function of Au further moves away from the valence band of the carbon nanotubes. Further research results show that DNA sensing is dominated by the diversity of the metal-SWNT connections rather than the channel conductance [113].

However, the carbon nanotubes prepared using this equipment are usually irregularly shaped and attached to the surface of SiO_2_, which will greatly reduce the transmission rate of the conductive channel and affect the electrical properties of the carbon nanotubes [125]. To solve this problem, Zhang et al. [126] prepared SWNTs suspended between palladium electrodes and arranged neatly in a high-temperature annealing treatment based on the dynamic motion of the silver liquid. Sun et al. [115] prepared CNT-based suspension FET sensors for the ultrasensitive detection of the DNA hybridization, as shown in Figure 5e. Compared with the unsuspended CNT-FET, the entire surface of the CNT can bind to DNA molecules, which significantly increases the sensing area of the FET. Moreover, silver nanoparticles on a SiO_2_ substrate have hydrophobic properties. When the silver film is heated to the melting point of 961 °C, the silver film in the channel becomes liquid and is infinitely mixed with Pd (melting point: 1552 °C), a constant force caused by the surface tension of 1.67 nN/μm is applied to the CNT during silver movement. Therefore, carbon nanotubes are straightened and suspended during such annealing. With the help of the surface tension of liquid silver, the CNT is suspended between two Pd electrodes during high-temperature annealing, which avoids the lack of direct attachment of the traditional FET-DNA sensor materials to the substrate, improves the ultrahigh carrier mobility and further enhances the performance of the FET-DNA sensor, as shown in Figure 5c. Subsequently, PBASE was used to combine DNA with a SWNT to obtain the ultra-high sensitivity detection of DNA. The detection limit (LOD) was as low as 10 aM, much lower than the previously reported CNT-FET sensor that attached the CNT to the substrate surface.

#### 2.2.2. Biosensors Based on the CNT-FET for Protein Detection

Label-free biosensors have attracted much attention due to their simple procedures, high sensitivity, rapid detection, easy miniaturization and integration. CNTs with a one-dimensional nanostructure have a strong sensitivity to the surface adsorption of many chemical substances and biological molecules, making CNTs an ideal material for constructing label-free biosensors to detect proteins and transforming the reaction process between biological molecules into the measurable change process of the CNT conductance [127]. Therefore, the biological recognition on the SWNT surface, such as the interaction between protein and protein or between protein and DNA, can be monitored by the electrical measurement of the FET biosensor. Proteins are often strongly bound to carbon nanotubes by non-specific (NSB) adsorption. For example, Balavoine et al. [128] proved that streptavidin was tightly bound to the side wall of carbon nanotubes in a spiral manner during incubation. The protein biosensors based on carbon nanotube field-effect tubes in recent years are summarized in Table 2.

As mentioned above, the commonly used methods for the chemical modification of carbon nanotubes with biological molecules, including proteins or aptamers, are the covalent functional modification or non-covalent functional modification. The covalent modification of carbon nanotubes is often oxidized to produce free carboxyl groups coupled with amino groups in proteins. Compared with the covalent modification of carbon nanotubes, the non-covalent modification method can retain the carbon nanotubes’ main structure and unique properties. This method mainly combines some substances as biomolecule joints with carbon nanotubes, then combines the biomolecules combined with the substances to be tested with the joints, and then detects the biomolecules to be tested. Chen et al. [144] reported the two-step hybrid modification of SWNTs and protein: firstly, 1-pyrenebutyrate succinimide ester was irreversibly adsorbed onto a SWNT through the π-π stacking interaction; then, the amino groups on the protein reacted with N-hydroxysuccinimide (PBASE) to form amide bonds through a nucleophilic substitution reaction, and the protein was immobilized on carbon nanotubes. Mazin A. et al. [136] uniformly coated a CNT on Si/SiO_2_ and then immobilized the SARS-CoV-2 S1 antibody on the CNT surface between the SD channel regions by a non-covalent interaction with PBASE. The electrical output of the CNT-FET biosensor was detected by introducing the SARS-CoV-2 S1 antigen.

**Figure 6:**
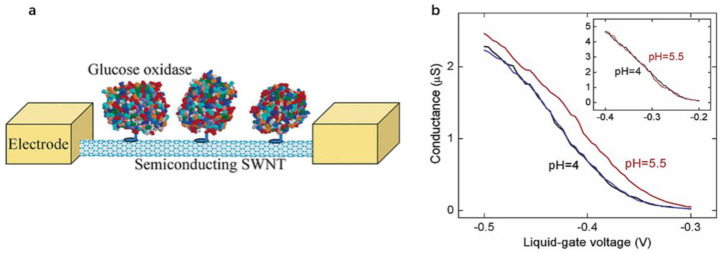
(**a**) semiconductor carbon nanotube device immobilized GOx enzyme; (**b**) the pH sensitivity diagram of the carbon nanotube sensor. At pH = 4.0 ± 0.2 (black line), then at pH = 5.5 ± 0.2 (red line), and finally at pH = 4.0 ± 0.2, the conductance of the semiconductor SWNT fixed by GOx used as the function of the liquid gate voltage (blue line). Reproduced from Koen et al. [145] by permission of the American Chemical Society.

Similarly, Alexander et al. [131] prepared CaptAvidin (an antibiotic protein modified with tyrosine) with a high sensitivity and selectivity by using 1-pyrenebutyrate succinimide ester as a connector to combine with biotin non-covalently. Dekker et al. [145] developed a CNT-FET protein biosensor made of a single SWNT and applied a variable electrolyte gate voltage through a platinum electrode. The device covalently coupled glucose oxidase (GOx) to the side wall of the SWNT via 1-pyrene succinimide ester, as shown in Figure 6a. GOx was fixed on the side wall of the semiconductor SWNT to reduce the conductivity of the tube. The enzyme layer on the SWNT could also inhibit the ions in the liquid close to the carbon nanotubes, making the pH of the device have a certain dependence, thereby further reducing the capacitance of the carbon nanotubes shown in Figure 6b.

However, this method will inevitably lead to the non-specific adsorption of proteins on carbon nanotubes, affecting the detection results. In response to this problem, Chen et al. coated a CNT by non-covalent binding of polyethene glycol (PEG) polymers (Tween 20). PEG coating made the CNT highly hydrophilic and charge-neutral, thus hindering the electrostatic binding and hydrophobic interaction with proteins [146]. Similarly, Star et al. [147] also fixed the biological molecules on SWNTs with a PEG/PEI coating to prepare the biosensors with high sensitivity and specificity, avoiding the nonspecific adsorption of proteins. Dai et al. [109] reported a non-covalent binding method to prepare CNT protein biosensors. Polymers such as Tween 20 or triblock copolymer chains can be irreversibly adsorbed on the nanotubes as a linker between the target biological molecules and the inhibitor of protein NSB. The CNT sensor, with this method, can specifically recognize and bind the target protein by binding the specific receptor of the target protein to the polyethene oxide functionalized nanotubes. 

Although there are still many biosensors for detecting proteins using the antigen-antibody reaction, the biosensors for detecting proteins in the SWNT-FET have also been developed for a long time. For example, aptamers with a high affinity and selectivity for multiple targets such as peptides, proteins and even whole cells, can compete with antibodies in a biological analysis. Lee et al. [130] used a thrombin DNA aptamer as a molecular recognition element. They immobilized it on the side wall of carbon nanotransistors by carbodiimidazole-activated Tween 20 (CDI-Tween) to form a thrombin aptamer-functionalized SWNT-FET to detect the thrombin molecules with a high sensitivity and high selectivity. 

**Figure 7:**
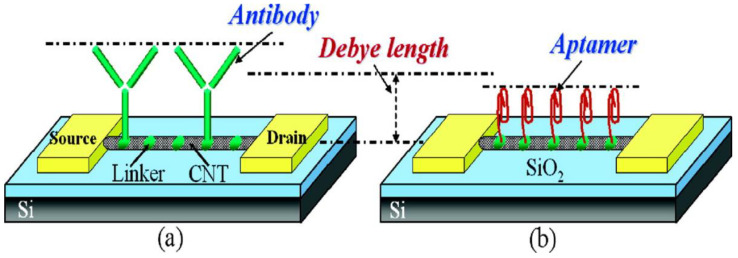
A label-free protein biosensor based on a CNT-FET: (**a**) antibody-modified CNT-FET; (**b**) Aptamer-modified CNT–FET. Reproduced from Kenzo et al. [134] by permission of the American Chemical Society.

Especially in the electrolyte-gated SWNT-FET biosensors, the nucleic acid aptamers have obvious advantages over the traditional antibody-based reagents. Firstly, aptamers can be chemically synthesized and stable in long-term storage, while antibodies are usually produced in organisms. Secondly, the size of the aptamer is less than the Debye length (Debye length is defined as the typical distance needed to screen the remaining charge of the moving carrier in the material) [148]. If the biomolecule is placed at a Debye length from the charge, its effect on the moving charge of the material is no longer considered. The standard size of the antibodies usually varies between 10 nm and 15 nm, much larger than the Debye length required in analytical buffer solutions [149]. Maehashi et al. [134] prepared label-free protein biosensors for detecting immunoglobulin E (IgE) based on the aptamer-modified carbon nanotube field-effect transistor (CNT-FET). The electrical properties of the CNT-FET were monitored in real-time by covalently fixing the 5′amino-modified aptamer on the CNT channel. The net source and leakage current of the aptamer-modified CNT-FET before and after the introduction of IgE increased with the increase of the IgE concentration. The detection limit of 250 pM was obtained. Its sensitivity was much higher than that of the sensor based on the antigen-antibody interaction. At the same time, it was proved that compared with the anti-IgE monoclonal antibody (IgE-mAb) with the size of 10 nm to 15 nm, it was easy to exceed the Debye length after binding to IgE, while the binding to the aptamer was less than the Debye length, as shown in Figure 7. It indicated that the aptamer-modified SWNT-FET had a better detection effect than the IgE-mAb-modified SWNT-FET under similar conditions. 

In addition, aptamers can be modified further to improve the sensitivity and selectivity of the sensor. Pawan et al. [133] prepared a novel synthetic receptor sensor based on the biomolecular recognition elements and molecular imprinting fusion to overcome some limitations of the traditional protein imprinting. The DNA aptamer with a specific affinity to the prostate-specific antigen (PSA) and thiolated modification was combined with PSA and then fixed on the gold electrode to control the electropolymerization of dopamine around the complex. In this way, aptamers can be fixed or closed to their binding conformation, and the PSA binding sites can be located on the sensor’s surface. Following the removal of the PSA, the molecularly imprinted polymer (MIP) cavity will cooperate with the embedded aptamer to form a hybrid receptor (apta-MIP), which improves the recognition more than a single aptamer. The detection limit of 1 pg/mL is three times higher than a single aptamer for the PSA detection. 

#### 2.2.3. Biosensors Based on the CNT-FET for Cell Detection

Conventional methods for monitoring the receptor activity include the radioactive binding assay, the luminescence method and the electrophysiological technique. However, these technologies have their limitations. For example, the binding assay requires a time-consuming preparation procedure. Some optical methods, such as surface plasmon resonance, can only be used to separate the receptor proteins or enzymes, but not for the whole cell. However, biosensors based on field-effect transistors (FETs) have been used to monitor the activities of various biological molecules, showing the advantages of simple, rapid and label-free detection. Therefore, the CNT can also be functionalized with specific antibodies to detect different cells, such as bacteria, pathogenic yeast or mammalian cells. Living cells can also be adsorbed on the surface of the carbon nanotubes due to physical or chemical reasons. Carbon nanotubes can also penetrate the cell membrane so that the cytoplasm of the cells can contact the FET so that the intracellular, transmembrane potential can be recorded. The detection becomes possible [150]. Villamiza et al. [151] prepared a field-effect transistor (FET) biosensor for the selective determination of Salmonella infant in formula by adsorbing the anti-Salmonella antibody onto single-walled carbon nanotubes as a conducting channel and coating the SWCNT with Tween 20 to prevent the non-specific binding of other bacteria or proteins. Sakaguchi et al. used a field-effect transistor (FET) biosensor to monitor the invasion process of cancer cells into the vascular endothelium in real-time [152] and an ion-sensitive field-effect transistor (ISFET) to monitor the respiration of cancer cells and normal cells in real-time [153].

**Figure 8:**
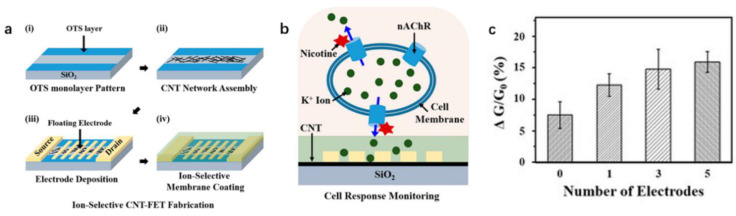
The schematic diagram describes the procedures for preparing sensors and measuring potassium ions released by living cells. Reproduced from Youngtak et al. [154] by permission of Molecular Diversity Preservation International. (**a**) Fabrication of the ion-selective carbon nanotube field-effect transistor (CNT-FET): (**i**) Patternization of the octadecyl trichlorosilane (OTS) layer; (**ii**) Specific adhesion of the carbon nanotubes; (**iii**) Dynamic electrode deposition on the CNT channel; (**iv**) Ion-selective membrane coating. (**b**) Direct monitoring of the response of a single cell to nicotine using ion-selective sensors. (**c**) Conductivity sensitivity of sensors with different numbers of floating electrodes to a 10 nM potassium ion solution.

However, the FET-based ion sensors are usually less sensitive. They can only be used to monitor the ion channel activity near a single cell, which has some limitations in the statistical analysis of multiple cells. In order to solve this problem, Zhao et al. [154] prepared a carbon nanotube field-effect transistor (CNT-FET) ion-selective sensor based on a floating electrode for the quantitative monitoring of nAChR in living cells. The device coated the potassium ion-selective membrane on the CNT-FET based on the floating electrode. The sensor can selectively detect the potassium ion through the membrane, as shown in Figure 8a. Subsequently, the sensor was used to monitor the release of potassium ions in a single PC12 cell stimulated by nicotine in real-time, as shown in Figure 8b. However, due to the Schottky barrier modulation, sensors with more electrodes exhibit larger sensor signals, i.e., the increase in the number of floating electrodes leads to an increased sensitivity of CNT-FET sensors [155], as shown in Figure 8c. The reason is that when the floating electrode is fabricated on the CNT channel, the Schottky barrier is formed at the interface between the semiconductor CNT and the metal electrode, and the height of the Schottky barrier is affected by the electrode work function. Therefore, the combination of the target molecules with the different electrode work functions can change the height of the Schottky barrier, resulting in the change of the channel conductance of the sensor and then detecting the target molecule. The increase in the number of Schottky barriers is the main factor determining the sensitivity enhancement of the sensor, independent of the shape or area of the floating electrode [156]. 

**Figure 9:**
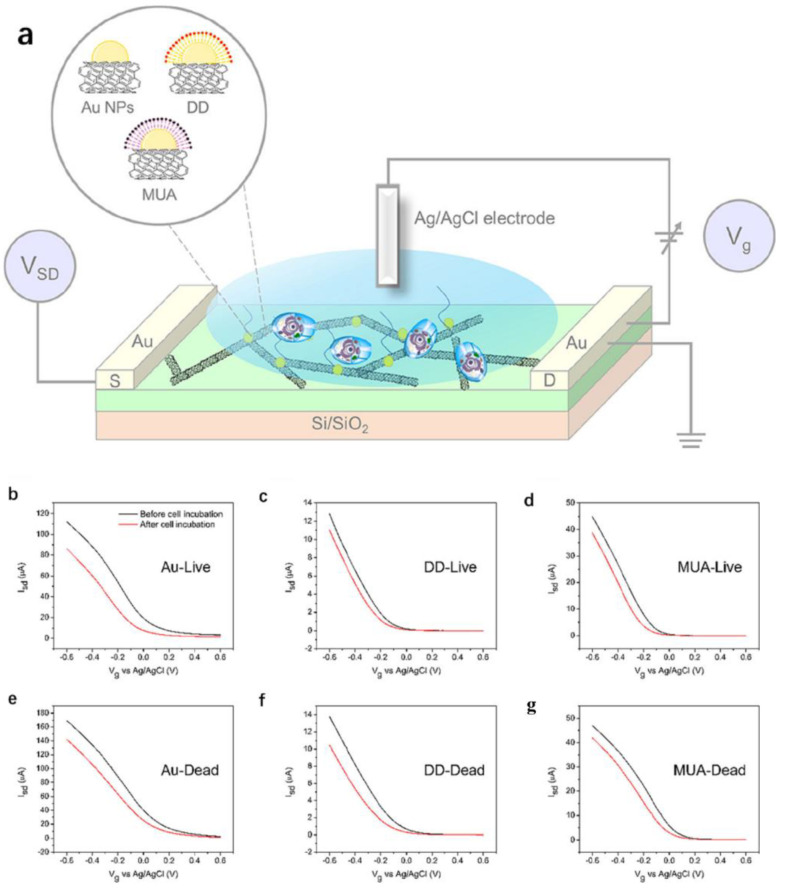
(**a**) The transmission characteristics of the CNT-FET, i.e., the relationship between the source-drain current (Isd) and the applied liquid gate voltage, have different decorative and liquid-gated NTFET schematics after the cell culture; (**b**) The transfer characteristics of NTFET were modified with bare gold nanoparticles; (**c**) dodecyl mercaptan; (**d**) 11-mercaptoundecanoic acid after the incubation in the living cells; (**e**) Transfer characteristics of NTFET after the incubation with bare gold nanoparticles; (**f**) 1-dodecanethiol; (**g**) 11-mercaptoundecanoic acid in dead cells. Reproduced from Liu et al. [157] by permission of the American Chemical Society.

In addition, the cell recognition based on the detection of specific target biomolecules usually requires additional labelling steps, and cell recognition may be hampered by the complexity of the biological matrix and the lack of clear target biomolecules. Studies have shown that the introduction of machine research methods can provide a new platform for cell recognition applications. Liu et al. [157] constructed a carbon nanotube field-effect transistor sensor array for a label-free whole-cell sensing. The array was based on three types of carbon nanotube field-effect transistors; namely, gold nanoparticles modified and gold nanoparticles modified semiconductor SWCNTs by a self-assembled monolayer (SAM) of dodecyl mercaptan (DD) and 11-mercaptoundecanoic acid (MUA) due to their respective hydrophobicity and hydrophilicity, as shown in Figure 9a. Different characteristics were extracted from the transfer characteristic curve, as shown in Figure 9b–g. Compared with living cells, the dead cells show changes in morphology, permeability and metabolism, which are expected to affect their interaction with the carbon nanotubes, resulting in different NTFET characteristics. The accuracy of the detection results can reach 87.5–93.8%, indicating that the constructed model can well identify living and dead cell samples. Guilherme et al. [158] also prepared arrays formed by metal nanoparticles functionalized decorated NTFET devices and successfully detected and distinguished malignant and non-malignant tissues and cells by extracting the selected NTFET feature transfer curve. 

#### 2.2.4. Chemical Sensors Based on the CNT-FET for Gas Detection

Because almost all atoms of carbon nanotubes can be exposed to the gas environment, they can provide a high specific surface area for gas adsorption, which is helpful to greatly improve the response sensitivity of the sensor. Therefore, gas sensors based on the CNT-FET have become the ideal choice for detecting water vapor, NO_2_, NH_3_, H_2_, H_2_S, C_2_H_5_OH, methanol vapor and other toxic gases or organic vapor molecules [159]. Whether a single carbon nanotube or a thin film of carbon nanotube field-effect transistor gas chemosensor, the sensing mechanism mainly comes from the gas adsorption on the CNT or metal, compared with the polycrystalline material such as metal oxide, carbon nanotubes. Their ability to prevent the sensor pollution improve the device’s long-term stability and anti-noise ability. The detection accuracy is also significantly better than that of traditional gas sensors and even the detection of single gas molecules has been widely studied [160,161,162,163,164]. The gas chemosensors, based on the carbon nanotube field-effect transistor in recent years are summarized in Table 3.

The study of carbon nanotube gas sensors began with the chemical sensors based on single-walled carbon nanotubes (SWNTs) reported by Kong et al. in 2000. Once the device was exposed to gaseous molecules such as NO_2_ or NH_3_, the resistance of the semiconductor SWNTs would increase or decrease sharply [178]. Tans et al. [179] studied the gas sensitivity of a CNT-FET to NH_3_ and NO_2_ and further explained the phenomenon; that is, when NH_3_ is detected, the Fermi level in the *p*-type CNT-FET moves to the conduction band, resulting in the decrease of hole concentration and the decrease of conductivity; when NO_2_ is detected, the Fermi level moves to the valence band, and the hole concentration and conductance increase. In addition, when the same gas concentration is different, the transfer curves also show relative differences. However, the detection of gas only through the adsorption of carbon nanotubes is far from the needs of human beings. Zhao [180] pointed out that NO_2_ and NH_3_ gases strongly interact with carbon nanotubes (chemical adsorption).

In contrast, other gases with a low adsorption capacity can only combine with carbon nanotubes (physical adsorption) through the van der Waals force. Woods [181] pointed out that the interaction between the volatile organic compound (VOC) molecules and CNTs was weak, which inhibited the performance of the gas sensors. Therefore, it is necessary to improve the reactivity of CNTs in monitoring volatile organic compounds (VOCs). Therefore, the sensitivity of the sensor detection is improved by the covalent or non-covalent functional modification of the carbon nanotubes with external substances, such as gold nanoparticles, specific solutions, polymers, metals or other substances. 

**Figure 10:**
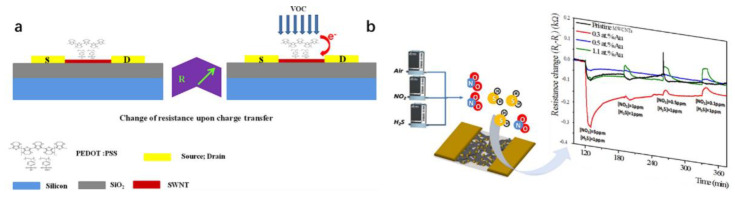
(**a**) schematic diagram of the VOC detection by a single-walled carbon nanotube gas sensor coated with polymer. Reproduced from Sushmee et al. [172] by permission of Elsevier Science Ltd.; (**b**) Different degrees of gold nanoparticles modified the multi-walled carbon nanotube gas sensor detection gas schematic diagram. Reproduced from Palmisano et al. [76] by permission of Elsevier Science Ltd.

The functionalized CNT-FET sensors usually provide a higher sensitivity and better selectivity than the original CNT-FET sensors. Slobodian et al. [182] used acidic KMnO_4_ to treat MWCNT, and the detection sensitivity of methanol gas increased by about 12–46% after the reaction. Sattarie et al. [170] spin-coated MWCNT and polyaniline (PANI) composites on glass and silicon substrates to detect methane gas. Compared with the single PANI film, the sensitivity of the MWCNT-PANI composite film to methane gas is significantly improved. Badhulika et al. [172] used a SWCNT with poly (3,4-ethylene dioxythiophene) polystyrene sulfonate (PEDOT: PSS) coating to detect the volatile organic compounds (VOCs), methanol, ethanol and methyl ethyl ketone. The detection limits were 1.3%, 5.95% and 3%, respectively, as shown in Figure 10a.

As a non-covalent functionalization method, the CNT and polymer composite nanomaterials do not destroy the physical properties of the CNT, which provides promising properties for sensing materials. An et al. [183] fabricated a gas sensor based on a SWNT and polypyrrole (PPy) nanocomposites, formed by spin coating the nanocomposites on the prefabricated electrode. The nanocomposites are about ten times more sensitive than the carbon nanotubes alone. Abraham et al. [184] fabricated a gas sensor using a composite film composed of multi-walled carbon nanotubes (MWNT) and PMMA. The device, fabricated by coating the composite film on a cross-finger electrode, shows a rapid response and an order of magnitude resistivity change, which can be used to detect dichloromethane, acetone and chloroform.

The gold nanoparticles functionalized by the CNT-FET gas sensors and prepared by E Dilonardo et al. [171] have high sensitivity and selectivity for NO_2_ and H_2_S, as shown in Figure 10b. SJ Young et al. [173] vaporized a 10 nm thick Fe layer on the synthesized CNT, and the prepared carbon nanotube ethanol gas sensor can reach 1.67% sensitivity. E. Radouane et al. [174] used a tin oxide modified MWCNT to detect 1 ppm nitrogen and 20 ppm carbon monoxide. This hybrid sensor has an excellent sensitivity and significantly eliminates cross-sensitivity to water.

QI et al. [185] proved that the non-covalent drop of polyethyleneimine (PEI) and Nafion (a polymerized perfluorosulfonic acid ionomer) onto a SWNT-FET would lead to a higher sensitivity and selectivity of gas sensors to NO_2_ and NH_3_. Following the PEI modification, the SWNT was changed from a *p*-type semiconductor to a n-type semiconductor. The sensor could detect less than 1 ppb NO_2_, but it was resistant to NH_3_ and was no longer sensitive. Compared with the sensor coated with PEI, the Nafion coating can make a SWNT insensitive to NO_2_ and show good sensitivity to NH_3_. Similarly, Star et al. [186] also used PEI to treat carbon nanotubes to prepare NTFET with PEI and a starch polymer coating. When the device was exposed to CO_2_ gas in the air at room temperature, its conductivity changed.

For the detection of H_2_, due to the lack of specific interaction between the bare CNT and H_2_, the carbon nanotubes also need to be processed. Molecularly specific nanotube sensors for H_2_ can be obtained by reasonable chemical and/or physical modifications of the nanotubes [187]. Kong et al. reported the sensitivity of a Pd-modified SWNT to H_2_ gas at ppm-level. The Pd nanoparticles coating was formed on the surface of the SWNT device by electron beam evaporation, and the conductivity decreased when the Pd-modified device was exposed to H_2_. The sensing mechanism is that H_2_ is decomposed into hydrogen atoms on the surface of palladium, which reduces the work function of palladium and the single-walled carbon nanotubes obtain electrons. Kim et al. [188] also used palladium modified carbon nanotube devices to prepare carbon nanotube sensors with a palladium layer, sensitively detecting the H_2_ concentration. In addition, Adarsh Kaniyoor et al. [189] prepared multi-walled carbon nanotubes functionalized with the rare metal Pt by droplet casting technology, which can detect hydrogen in the air with 4% vol hydrogen content that can stably exist in the repeated cycles of hydrogenation and dehydrogenation. 

**Figure 11:**
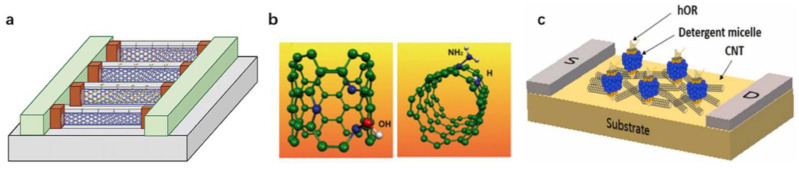
(**a**) Structure diagram of the doped carbon nanotubes. Reproduced from Shu et al. [191] by permission of of the American Chemical Society; (**b**) Molecular model of the carbon nanotube, whose surface is doped with OH group (red) and NH_2_ group (blue) with pyridine site. Reproduced from Villalpando—Páez et al. [190] by permission of Elsevier Science Ltd.; (**c**) Schemata of the hOR-based CNT-FET sensors for the DMMP detection. Reproduced from Jin et al. [176] by permission of Elsevier Science Ltd.

In addition, impurity atoms such as pyridine-like sites, boron atoms and nitrogen atoms can also be added to carbon nanotubes to improve their gas sensing properties. Villalpando-Páez F. [190] reported an ordered CNx nanotube sensor due to the presence of highly reactive pyridine-like sites on the tube surface, as shown in Figure 11a. It can bind strongly to ammonia, acetone and OH groups, thus sensing toxic substances and further altering their density of states. This type of sensor is not only responsive but also reusable. Peng S. [191] reported that a SWCNT was entered by doping impurity atoms (such as boron and nitrogen atoms), as shown in Figure 11b. The molecular adsorption on the modified OH groups and NH_2_ can overcome the problems caused by the weak van der Waals interaction between the single-walled carbon nanotubes and doped materials.

Toxic organic chemicals can cause harm to human health and the environment. These chemicals often exist in liquid form, but their vapor can enter the human body through the skin and respiratory tract. A rapid and accurate detection of these chemicals is essential to protect human health. Dimethyl methylphosphonate (DMMP) is a structural mimic of the chemical warfare agent (CWA) Salin. Salin is a representative of the neurotoxic CWAs and an organophosphorus toxin. It can deactivate acetylcholinesterase in the human body, making it difficult for the cholinergic synapses to control neurotransmitters, paralyze neurons in the body and lead to death [192]. Jin et al. [176] used a single-walled carbon nanotube field-effect transistor (SWNT-FET) based on the human olfactory receptor (hOR) as a platform for detecting DMMP, as shown in Figure 11c. Because hORs are highly specific to some of their target molecules, the biological signals of hORs are converted into highly sensitive electrical signals by the SWCNT-FET, and then the hOR2T7 with a high selectivity to DMMP is selected for the development of the hOR2T7 B-nose. The hOR2T7 B-nose can selectively detect DMMP at 10 fM concentration.

Similarly, Novak et al. [193] also prepared a SWNT-FET sensor to detect dimethyl methyl phosphonate (DMMP). The device is reversible and can detect DMMP at a sub ppb concentration level (GS = 0 V) under the influence of hydrocarbon vapor and certain humidity. Following the detection, the bias voltage of +3 V applied to the gate can cause the Coulomb interaction between the negative charge and DMMP as a strong electron donor, which can be recovered quickly in a few minutes. Based on this principle, Chang et al. [194] used the negative back gate voltage to recover the NH_3_-contacted SWNT-FET sensor and the positive voltage to recover the NO_2_-contacted SWNT-FET sensor. At the same time, this effect can also be used to partially avoid the low induction specificity of the original SWNT sensor and better identify the analyte. 

#### 2.2.5. Chemical Sensors Based on a CNT-FET for Ion Detection

The dysregulation of specific ion levels in an organism or environment is known to have adverse effects on human health and the environment. It is crucial to be able to quickly, sensitively, constantly and stably detect concentrations of certain ions due to the role of ions in pathophysiology and the high demand for sensitive and selective methods to identify these species in biological systems. Among the sensors available for ion detection, field-effect transistor (FET) sensors demonstrate great potential for rapid and real-time detection. Due to their features like a fast electron transfer, a large specific surface area, good electrical and thermal conductivities and corrosion resistance, carbon nanotubes have emerged as a suitable sensor material for ion detection. Carbon nanotube field-effect tube chemosensors for ion detection in recent years are summarized in Table 4.

The RNA-cut deoxyribonuclease is usually used for the sensitive and selective HMI detection in chemosensors or chemical sensors. The metal-dependent DNAzyme has unique chemical properties and can bind to specific metal ions, making it an ideal recognition element [206]. The DNAzyme is usually composed of an enzyme chain and substrate chain, in which the substrate chain contains a single ribonucleic base (RNA) bond as the cleavage site. These two chains are hybridized in a buffer solution to form a double helix in the absence of target metal ions. When the target metal ions appear, the DNAzyme cuts the substrate chain and releases short fragments from the double-strand. The changes in the structure of the DNAzyme will lead to changes in the number of carriers in carbon nanotubes. Therefore, the DNAzyme is very convenient for the FET sensing probe to detect the concentration of metal ions [207,208]. 

**Figure 12:**
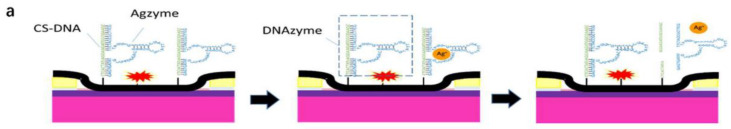

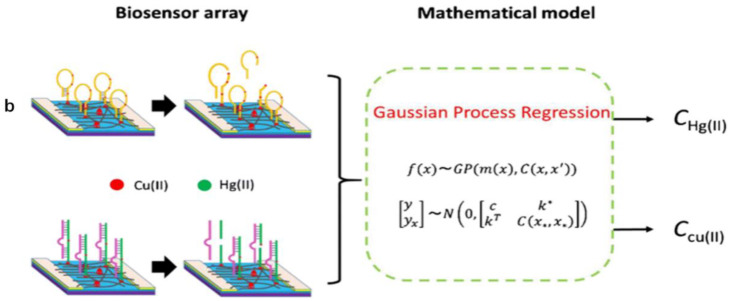
(**a**) DNAzyme cut its substrate chain at the RNA site in the presence of Ag^+^. Reproduced from Yang et al. [197] by permission of Elsevier Science Ltd.; (**b**) DNAzyme cleavage of its substrate chain at the RNA sites in the presence of Cu^2+^ and Hg^2+^ and the Gaussian process regression formula. Reproduced from Hui et al. [209] by permission of Elsevier Science Ltd.

Wang et al. [195] used a silver-specific RNA-cut DNAzyme modified carbon nanotube field-effect transistor chemosensor to detect Ag (I). The Agzyme binds to the complementary DNA (CS-DNA) strand and covalently immobilizes the CS-DNA on the SWNT, as seen in Figure 12a. In the presence of Ag^+^, the substrate chain is cut into fragments and released from the RNA base, resulting in changes in the structure of the DNAzyme and thus changes in the conductivity of the CNT-FET. In addition, Wang et al. also used specific Pbzyme modified SWNTs to detect lead ions (Pb^2+^). The chemosensor used 3-aminopropyltriethoxysilane to fix the SWNTs in the area between the source electrode and the drain electrode of the single-gap microelectrode (FET), and the double-stranded DNA (Pbzyme) composed of the DNAzyme (GR-5) and complementary DNA (CS-DNA) was fixed on the surface of the SWNT using a peptide bond. Pb^2+^ can cut the CS-DNA to change the structure of the Pbzyme on the surface of the SWNTs, thereby affecting the number of carriers in the SWNTs and the conductivity of the carbon nanotubes to detect the Pb^2+^ concentration with a high sensitivity and selectivity.

In addition, Wang et al. [196] also used specific the Pbzyme modified SWNTs to detect lead ions (Pb^2+^). The chemosensor used 3-aminopropyltriethoxysilane to fix the SWNTs in the region between the source electrode and the drain electrode of the single-gap microelectrode (FET), and the double-stranded DNA (Pbzyme) composed of the DNAzyme (GR-5) and the complementary DNA (CS-DNA) was fixed on the surface of the SWNT using a peptide bond. Pb^2+^ can cut the CS-DNA to change the structure of the Pbzyme on the surface of the SWNTs, thereby affecting the number of carriers in the SWNTs and the conductivity of the carbon nanotubes to detect the Pb^2+^ concentration with a high sensitivity and selectivity. 

However, not all metal ions have metal-dependent DNAzymes that can specifically bind to them. Because Hg^2+^ and Cu^2+^ have a high sulfur affinity, both can form stable complexes with the DNAyzme, so this method cannot be used to detect Cu^2+^ when Hg^2+^ and Cu^2+^ coexist in the matrix. To solve this problem, Wang et al. [209] proposed a chemosensor based on the SWCNT-FET to determine Cu^2+^ and Hg^2+^ using the Gaussian process regression (GPR). The DNAzyme PSCu10 and its complementary DNA were inserted into the thiophosphate RNA (CS-DNA) for the functionalization. The CS-DNA containing a 5′ amino group was immobilized on the surface of the SWNTs using a peptide bond and then combined with PSCu10 using base pairing. When Cu (II) was bound to PSCu10, the CS-DNA (substrate chain) split at the RNA site, and the Gaussian process regression was used to establish a prediction model to estimate the concentration of Cu^2+^ [197]. In order to study the response of the sensor to the target Cu^2+^ and Hg^2+^, the Hgzyme (the mercury-specific DNAzyme) and PSCu10 were immobilized on SWCNT-FET sensors with their respective substrate chains. The sensor array was immersed in a solution with different concentrations of Cu^2+^ and Hg^2+^ ranging from 0.01 nM to 10,000 nM. The results show that, compared with the response of Hg^2+^, the percentage of the relative resistance increases with the increase of the ion concentration in the presence of Cu^2+^. The LODs of the sensor for Cu^2+^ and Hg^2+^ were 6.7 pM and 3.43 nM, respectively. The accuracy of Cu^2+^ concentration prediction was expressed by the correlation coefficient (R_0_) of 0.985 and the root means square error between the actual Cu^2+^ ion concentration and the predicted Cu^2+^ concentration of 0.038. Based on this method, Wang et al. [210] used similar sensing methods to prepare SWCNT-FET sensors for detecting and monitoring Cd^2+^ in feed. Similar to Cu^2+^, Cd^2+^ also lacks the specific DNAzyme, and the interference of Cd^2+^, Hg^2+^, Pb^2+^ and other metal ions will also hinder the recognition. Therefore, a carbon nanotube field-effect transistor chemosensor with three different types of DNAzyme (Cdzyme, Hgzyme and Pbzyme) and their respective substrate chains fixed on the channel surface was prepared. The Gaussian process regression was also used to collect the percentage of the resistance data. The detection results showed that the LOD was 34 pM. Although this study has successfully proved that the non-specific DNAzyme-functionalized single-walled carbon nanotube field-effect transistor chemosensor combined with the GPR prediction can be used as a sensor to identify the specific metal ions in complex solutions, more general methods need to be developed to achieve various sensing objectives. 

**Figure 13:**
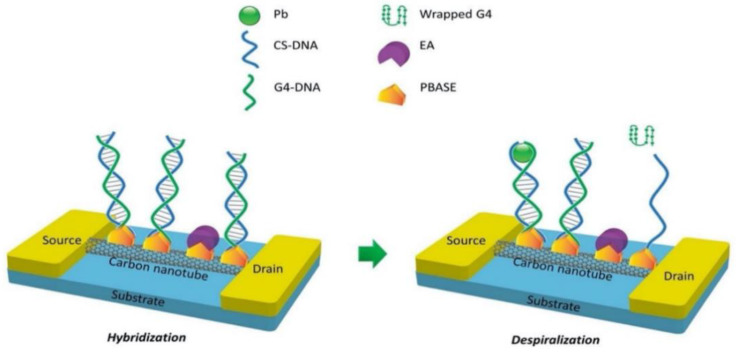
Hybridization and desalinization of CNT-FET sensors induced by G4-DNA and CS-DNA. Reproduced from Wang et al. [209] by permission of Elsevier Science Ltd.

Although the DNAzyme has a high sensitivity and selectivity for the specific HMI, its structure is destroyed during the sensing process (the substrate chain is cut at the RNA site). Therefore, the DNAzyme/FET sensor cannot be reused for long. However, the aptamer-modified CNT-FET studied the FET functionalized aptamer as an alternative probe for detecting HMI. Compared with the DNAzyme, aptamer (a single-stranded DNA) is better for the sensor recognition components because the aptamer changes its structure only when the target exists. Wang et al. [210] prepared a G-quadruplex aptamer (G4-DNA) and complementary CS-DNA functionalized FET chemosensor to determine Pb^2+^ ions, and can be reused. The G4-DNA is a functional DNA molecule with a specific binding affinity to Pb^2+^. The sensor mechanism is that Pb^2+^ ions can effectively induce the conformational change of the G4-DNA. In the absence of Pb^2+^, the G4-DNA and CS-DNA are hybridized to form double-stranded DNA (double-stranded DNA). However, in the presence of Pb^2+^, double-stranded DNA is dehelixed by Pb^2+^. As shown in Figure 13, the conformational change of the aptamer (hybridization and dehelicity) leads to a change in the electrical conductivity of SWCNTs, resulting in a decrease in resistivity, which is then used for Pb^2+^ detection. The sensor can range from 1 ng/L to 100 ng/L.

## 3. Conclusions and Future Prospects

Carbon nanotubes have become the core material of the new sensor architecture due to their structure and excellent electrical properties. In this paper, the latest progress in detecting biomarkers for biochemical sensors based on the CNT-FET is reviewed. The preparation method and physical and electronic properties of the CNT make it very suitable for the preparation of biochemical sensors. At the same time, the CNT can also be combined with other nanomaterials to form composite materials to enhance or utilize these properties. How to use different biomolecules to modify their covalent and non-covalent surface functionalization to produce sufficient sensitive and selective sensing signals. The preparation includes the general FET structure and electrolyte gated FET. The sensors based on the CNT-FET have been applied to monitor various gases effectively. At the same time, the functionalization of the biological receptors based on the CNT-FET can construct various biochemical sensors for the detection of various proteins, cells, nucleic acids and heavy metal ions. Since nano-channels with a large surface volume ratio and thin atomic bodies provide an ideal electrostatic control, small changes in the surrounding environment can significantly change the electrical characteristics of the CNT-FET, and small concentrations of a target analyte can also be combined with the surface of the channel to cause detectable changes in electrical signals. Therefore, these sensors can show a high sensitivity to the target analysis.

In a recent report, Lin et al. [211] used a DNA-directed guanine (G)-specific cross-linking chemical covalent modification of carbon nanotubes to maintain the modification site with the nanotube lattice. Through DNA screening, it was determined that the specific sequence could react with the enantiomers to produce the smallest disorder-induced Raman mode intensity and photoluminescence Stokes shift, indicating the formation of an ordered defect array. The reaction mechanism analysis shows that the spiral period is generated by a series of G-modified carbon-carbon bonds separated by a fixed distance along the armchair spiral line, which can be used to reshape the nanotube lattice to obtain new electronic properties. Zheng et al. [212] synthesized a one-dimensional (1D) van der Waals heterostructure in which the different atomic layers (e.g., boron nitride or molybdenum disulfide) were seamlessly wrapped around a single-walled carbon nanotube (SWCNT) and formed coaxial crystalline heterostructure nanotubes. Many internal structural details can be achieved without transfer and access, especially to distinguish the edges of boron nitride nanotubes (BNNT) with different shapes. These edges are confirmed to be closely related to their own chiral angles and polarity by electron diffraction at the same position. In addition, they elucidated the chiral correlation between SWCNT templates and BNNT crystals. This work not only makes an in-depth study of the one-dimensional heterostructure material group, but also can be used as an interesting study of crystal growth on highly curved (several nanometer radius) atomic substrates. These results demonstrate that the research progress of single-walled carbon nanotubes with a special chirality and heterostructures are good materials for the functional design of biosensors for the future.

Although great progress has been made in biochemical sensors based on carbon nanotube field-effect transistors for detecting cell properties, they are far from satisfying human needs compared with other biomarkers. We expect the sensor based on CNT transistors to deepen the basic understanding of the interaction between an analyte and the CNT-FET and explore its wide application in health care, environment and food. Although great progress has been made in CNT-FET based biochemical sensors, the field still faces key challenges in practical applications, including: (1) the unstable electrical properties of CNT-FETs over time. (2) Sensor performance fluctuations caused by small changes in the surrounding environment, such as buffer conditions, noise interference, etc. (3) Nonlinear calibration curve between the electrical signal and analyte concentration. These problems hinder the realization of portable and miniaturized systems using FET conversion. The coronavirus pandemic in the early 2020s will undoubtedly redouble research interest in areas such as rapid virus diagnostic techniques and bedside patient monitoring, as the World Health Service is adapting to the impact of the pandemic [213]. CNT-based analytical equipment will undoubtedly play an important role in addressing many of these challenges with its potential to develop biochemical sensors with a high sensitivity and fast response time.

## Figures and Tables

**Table 1:** DNA detection based on CNT-FET.

Analyte	Detection Limit	Functionalized Modification of Carbon Nanotubes	Detection Range	Reference
ssDNA	14 pM	/	1–200 nM	[111]
ssDNA	60 aM	Y_2_O_3_ film/AuNPs	100 aM–1 fM	[43]
ssDNA	Single-molecule	single point defects	/	[112]
ssDNA	1 pM	CNT aerogel	/	[113]
ssDNA	6.8 fM	AuNPs	/	[114]
ssDNA	10 aM	PBASE	10 aM–1 pM	[115]
ssDNA	0.1 nM	CNT-COOH	0.1–20 nM	[116]
Exosomal miRNA	0.87 aM	Y_2_O_3_ film/AuNPs	1 aM–1 nM	[117]
hepatitis B	<1 mM	AuNPs	10^−18^ M–10^−6^ M	[118]
papilloma virus	<1 mM	AuNPs	10^−18^ M–10^−12^ M	[118]
influenza virus	0.5 nM	CNT-COOH	1–10 nM	[69]
H5N1 virus	1.25 pM	CNT-COOH	1.0 pM–100 nM	[119]
avian influenza virus	1 EID 50/mL	/	6 × 10^2^–2 × 10^4^ EID 50/mL	[120]
(SIV) H1N1	180 TCID 50/ml	CNT-COOH	10^3^–10^5^ TCID 50/mL	[121]
Hepatitis C virus	0.7 fM	CNT-COOH	0.1 fM–1 pM	[122]
Adenosine	100 pM	PBASE	100 pM–10 µM	[65]

**Table 2:** Protein detection based on a CNT-FET.

Analyte	Detection Limit	Functionalized Modification of Carbon Nanotubes	Detection Range	Reference
DENV	8.4 × 10^2^ TCID_50_/mL	Heparin	/	[129]
Thrombin	10 nM	CDI-Tween	0–100 nM	[130]
CaptAvidin	/	Pyrene	/	[131]
Streptavidin,	1.47 nM	Pyrene	1.6 nM–1.6 μM	[132]
Prostate-specific antigen	1 pg/mL	/	100 pg/mL~100 ng/mL	[133]
IgE	250 pM	PBASE	250 pM–20 nM	[134]
Ara h1	/	PBASE	0.63–0.95 μg/mL	[135]
SARS-CoV-2 spike protein (S1)	4.12 fg/mL	PBASE	0.1 fg/mL–5.0 pg/mL	[136]
SARS-CoV-2 spike protein (S)	5.5 fg/mL	EDC/NHS	5.5 fg/mL–5.5 pg/mL	[137]
Antibodies of HbcAg	0.03 ng/mL	Hyaluronic acid	1–5 ng/mL	[138]
NS1 protein	12 ng/mL	CNT-COOH	40 ng/mL–2 µg/mL	[139]
Microvesicles	6 particles/mL	Y_2_O_3_ film/AuNPs	6 × 10^0^–6 × 10^6^ particles/μL	[43]
Prostate-specific antigen	84 pM	PBASE	500 pM–100 nM	[140]
BoNT	52 fM	PBASE	52 fM–500 fM	[141]
AQP4 antibody	1 ng/L	/	1 ng/L–1 µg/L	[142]
Cysteine	0.45 fM	CCD1	1 fM–1 nM	[143]

**Table 3:** Gas detection based on CNT-FET.

Analyte	Detection Limit	Functionalized Modification of Carbon Nanotubes	Detection Range	Reference
NO_2_	10 ppb	PDMS	100–1000 ppb	[161]
NO_2_	0.086 ppm	/	100 ppb–10 ppm	[165]
NO_2_	125 ppt		0.5–20 ppm	[166]
NH_3_	/	/	100–500 ppm	[163]
Acetone/Voc	/	Porphyrins	/	[164,165,167]
Cl_2_	1.33 ppb	Phthalocyanin/SWCNT-COOH	0.25–2 ppm	[168]
Cl_2_	0.27 ppb	F_16_ CuPc	0.1–2 ppm	[169]
CH_4_	/	PANI	/	[170]
Carbonyl Chloride	630 nm/RIU	/	/	[171]
NO_2_	0.1 ppm	Au NPs	0.1–10 ppm	[76]
Methanol	1.3%	PEDOT:PSS	2.5–75%	[172]
Ethanol	1.67%	/	50–800 ppm	[173]
Ethanol	5.95%	PEDOT:PSS	/	[172]
MEK	3%	PEDOT:PSS	/	[172]
CO	/	tin oxide nanoclusters	2–20 ppm	[174]
H_2_	20 ppm	Pt nanoparticle	20–200 ppm	[175]
DMMP	10 fM	hOR2T7	10^−16^–10^−7^ M	[176]
DMMP	2 ppb	/	2 ppb–2 ppm	[177]

**Table 4:** Ion detection based on CNT-FET.

Analyte	Detection Limit	Sensitivity	Detection Range	Reference
Ag^+^	5 pM	/	10 pM–1 μM	[195]
Pb^2+^	7.4 pM	/	10 pM–50 nM	[196]
Hg^2+^	3.43 nM	/	5 nM–10 μM	[197]
Cu^2+^	6.7 pM	/	10 pM–10 μM	[198]
pH	1 mM	7600 mV/pH 23%/pH	pH 3–10	[199]
pH	100 mM	/	pH 3–5	[103]
pH	10 mM	71 nA/pH 7.5%/pH	pH 2–7.5	[199]
pH	/	17 nA/pH 8.2%/pH	pH 3–8	[200]
pH	10 mM	3.9 µA/pH 13%/pH	pH 3.4~7.8	[201]
Hg^2+^	2 ppb	/	10 nM–1 mM	[202]
Cu^2+^	3 ppt	/	3~29 ppt	[203]
Ca^2+^	100 pM	69 nA	100 nM~1 mM	[204]
Ca^2+^	10^−15^ M	/	10^−15^–10^−13^ M	[205]
Cl^−^	0.6 µg·L^−1^	−446 nA·L·mg^−1^	/	[205]

## Data Availability

Data available on request due to restrictions e.g., privacy or ethical. The data presented in this study are available on request from the corresponding author.
